# Insights on the sonochemical synthesis, characterization, and effective lead (Pb^2+^) adsorption ability of nano lanthanum oxide (La_2_O_3_)

**DOI:** 10.1039/d6ra03226e

**Published:** 2026-08-20

**Authors:** Yana Bagbi, Enuk Libang, Srianshu Kumar Panda, Sanjeev Kumar, Narender K. Dhania

**Affiliations:** a Department of Physics and Astrophysics, University of Delhi Delhi 110007 India yanabagbi@gmail.com narender@zoology.du.ac.in; b Wound Healing Research Laboratory, Department of Zoology, Faculty of Science, University of Delhi Delhi 110007 India; c Department of Physics, Rajiv Gandhi University Itanagar Arunachal Pradesh 791112 India

## Abstract

Lead contamination in water sources due to industrial pollution, informal recycling, the use of leaded products, and mining is a severe public health crisis affecting millions, resulting in significant economic losses and health impacts that require urgent treatment strategies to address lead-contaminated water. The key innovation of this research is the surfactant-free sonochemical synthesis of non-agglomerated, highly active nano La_2_O_3_, designed for the rapid, sustainable, and highly recyclable removal of lead (Pb^2+^) from water. This synthesis method utilized acoustic cavitation, which results in the formation of dense surface oxygen vacancies and native hydroxyl (–OH) functional groups. The morphology, composition, and surface properties were studied using powder X-ray diffraction (XRD), transmission electron microscopy (TEM), scanning electron microscopy with energy-dispersive X-ray spectroscopy (SEM/EDX), and Fourier transform infrared (FTIR) spectroscopy. The average size of nano La_2_O_3_ was 15 nm, as obtained from XRD and TEM analyses. A surface charge of +22 mV was obtained using the zeta potential technique. Batch sorption experiments were conducted by varying the parameters such as the solution pH (2.0–7.0), agitation time (2–30 min), temperature (15–45 °C), and adsorbent dosage (0.1–0.5 g L^−1^). Almost 98% of Pb^2+^ ions were removed from a 100 mL solution containing 50 mg Pb^2+^ per L within 30 min. The experimental data of Pb^2+^ adsorption were fitted to the pseudo-first-order and pseudo-second-order kinetic models, and the pseudo-second-order kinetic model was the best-fitted model, indicating the chemisorption of Pb^2+^ ions onto nano La_2_O_3_. The rate of equilibrium adsorption capacity (*q*_e_, mg g^−1^) of lead uptake was obtained to be 19.12 mg g^−1^. Desorption studies were successfully performed for five cycles to regenerate the spent nano La_2_O_3_ using HNO_3_ (0.05 M) as the desorbing agent, thereby enabling stability and reuse.

## Abstract 2

Schematic of the synthesis of nano La_2_O_3_ and its application in the adsorption of aqueous Pb^2+^ ions.

## Graphical abstract

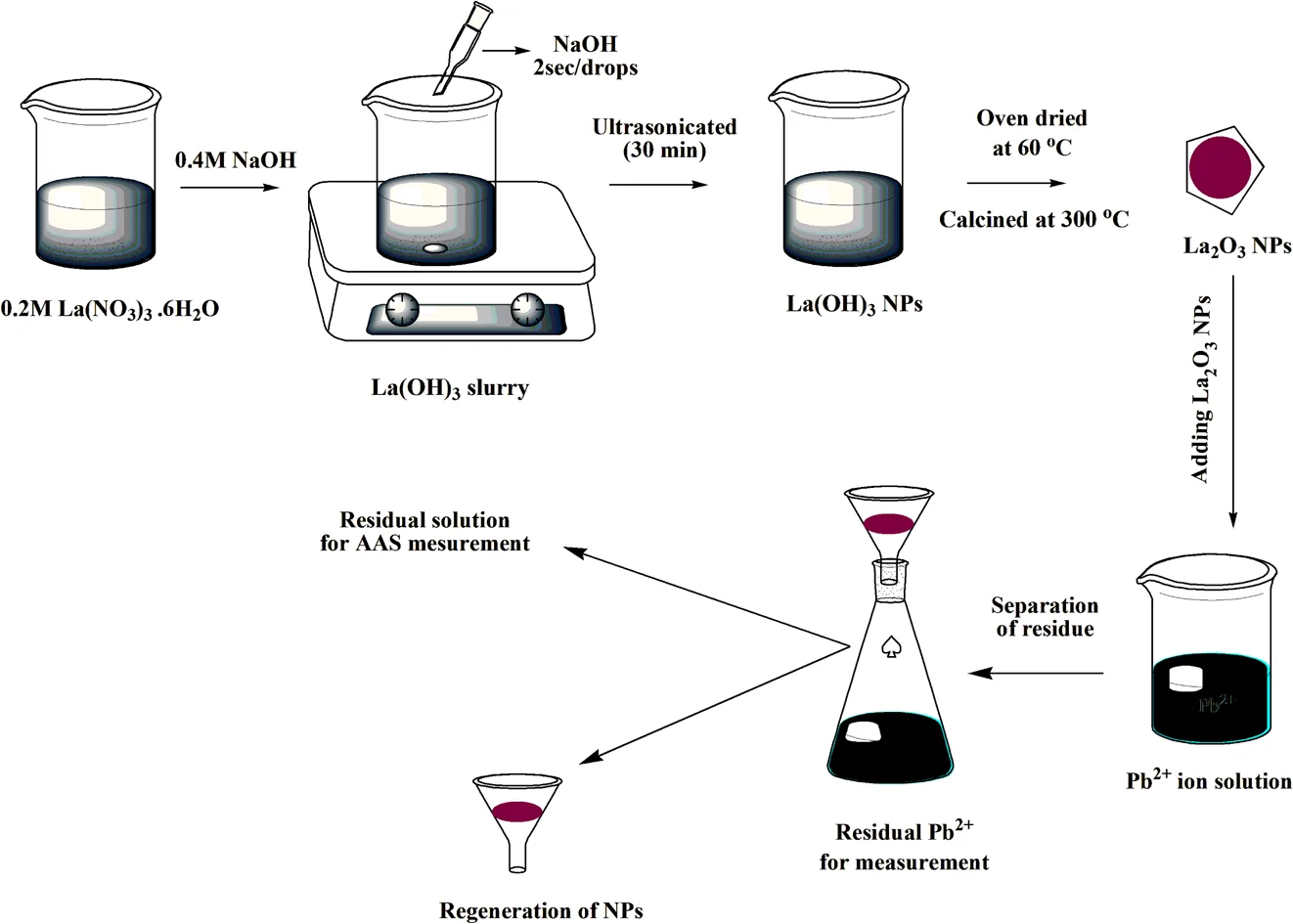

## 1 Introduction

Metal oxide nanoparticles, including nanosized iron oxides,^1,2^ manganese oxides,^3^ magnesium oxides,^4^ aluminium oxides,^5,6^ titanium oxides,^7^ and cerium oxides,^8,9^ have recently evoked great research interest for heavy metal adsorption from water. The large surface areas, cost-effectiveness, and high selectivity of metal oxide nanoparticles caused by the size quantization effect^10,11^ result in the deep removal of toxic metals from water.^11^ It has become a hot topic to develop new technologies for synthesizing metal oxide nanoparticles to remove heavy metals under varying experimental conditions and to reveal the underlying mechanism responsible for heavy metal removal.

It is well known that heavy metals are introduced into the environment both naturally and through industrial processes. They have high atomic weight and density, at least 5 times greater than those of water.^12,13^ Their multiple industrial, domestic, agricultural, medical, and technological applications have led to their wide environmental distribution, raising concerns over their potential effects on human health and the environment.^14–16^ Their toxicity depends on several factors, including the dose, route of exposure, chemical species, and age, gender, genetic background, and nutritional status of the exposed individuals.^12,15^ In particular, environmental contamination by lead is a significant problem in industrialized areas, especially those with a large number of electroplating and metal-finishing industries.^1,2^

The United States Environmental Protection Agency (USEPA) has set the maximum contaminant level (MCL) in drinking water at 0.010 mg L^−1^ for lead.^17^ The World Health Organization (WHO) and the Bureau of Indian Standards (BIS) have set the maximum permissible limit of lead in drinking water at 0.015 mg L^−1^, respectively.^18,19^ Several techniques have been introduced to remove toxic heavy metals, including lead (Pb^2+^), from water, such as chemical precipitation,^20^ ion exchange,^21^ reverse osmosis,^22^ membrane filtration^23^ and adsorption.^24^ Among these methods, adsorption is a highly efficient, recyclable, and economical removal technique.^1,2^ Consequently, there is an urgent demand for economical, highly effective, and reliable adsorbents capable of reducing lead levels to the applicable regulatory level in water. Different types of nano-adsorbents, such as activated carbons,^25^ clay minerals,^24^ and chitosan/natural zeolites,^26^ have been researched to adsorb heavy metal ions, such as lead (Pb), arsenic (As), cadmium (Cd), chromium (Cr), and mercury (Hg), from aqueous solutions. However, the application of these materials has been limited due to their low adsorption capacity and small surface areas. Many researchers have used iron oxide nanoparticles to adsorb metal ions. However, their application is limited due to their poor stability and surface aggregation.^1,2^ Thus, in this study, a simple, economical, and non-toxic synthesis route for lanthanum oxide nanoparticles was attempted; these nanoparticles exhibited high biocompatibility and selectivity for lead ions. Lanthanum oxide has a higher sorption capacity than activated alumina due to its higher isoelectric point (IEP) of 11.1 compared with that of the 9.2 of activated alumina.^27^ Lanthanum is extracted from bastnaesite and monazite^28^ and is one of the cheapest rare-earth elements. Lanthanum oxide is also known to be nontoxic and environmentally friendly.^27,29^ Adutwum^30^ reported 30–40 mg (Se)/g adsorption capacities from water using lanthanum oxide and binary lanthanum–aluminum oxide. Wasay *et al.*^31,32^ reported the removal of selenite, fluoride, phosphate, and arsenate ions from water using the adsorption process of lanthanum-impregnated silica gel and lanthanum-impregnated alumina. In addition, lanthanides have unique 4f electronic configurations, which have been used in various applications; materials based on lanthanides have attractive and exciting optical, electrical, magnetic, and therapeutic properties.^31,34^

Even though many methods, including hydrothermal,^36^ solvothermal,^37^ and microemulsion- or reverse micelle-based methods,^38^ have been developed for the synthesis of lanthanum nanostructures, some of these methods require long reaction times, high temperatures, high pressures, and costly raw materials. In 1999, a simple, effective, and innovative approach known as the sonochemical method was developed for preparing lanthanum oxide nanostructures.^39,40,45^

In this study, nano lanthanum oxides were synthesized using a sonochemical reaction using lanthanum acetate and sodium hydroxide (NaOH) as a reducing agent to effectively remove lead from water. The key innovation of this work is the surfactant-free sonochemical synthesis of non-agglomerated, highly active nano La_2_O_3_, designed for the rapid, sustainable, and highly recyclable removal of lead (Pb^2+^) from water. This synthesis method utilizes acoustic cavitation, which results in the formation of dense surface oxygen vacancies and native hydroxyl (–OH) functional groups. Nano La_2_O_3_ exhibited rapid lead adsorption kinetics, reaching equilibrium within 25 minutes under optimized conditions at solution pH 6, 25 °C, and an initial Pb^2+^ concentration of 50 ppm. The surface morphology, composition, and properties of the nanoparticles were analyzed using various techniques, including powder X-ray diffraction (XRD), transmission electron microscopy (TEM), scanning electron microscopy with energy-dispersive X-ray spectroscopy (SEM/EDX), and Fourier transform infrared (FTIR) spectroscopy. Biocompatibility studies of nano La_2_O_3_ were also conducted. The point of zero charge pH (pH_PZC_) for nano La_2_O_3_ was obtained at a solution pH of 5. Batch sorption analysis was performed by varying the solution pH (ranging from 2.0 to 7.0), agitation time (from 2 to 30 minutes), temperature (between 15 °C and 45 °C), and adsorbent dosage (from 0.1 to 0.5 g L^−1^). Additionally, sorption dynamic studies were carried out, and a potential mechanism for the adsorption of Pb^2+^ ions on nano La_2_O_3_ was discussed in detail. Regeneration studies of the spent nano La_2_O_3_ were also conducted through five cycles of desorption.

## 2 Experimental

### 2.1 Materials and methods

All chemical reagents, such as lanthanum nitrate (LaN_3_O_9_·6H_2_O) (99.9%) and lead nitrate Pb(NO_3_)_2_ (99%), were of analytical grade and purchased from Sigma-Aldrich, USA. Ammonia solution (NH_3_, 30%), hydrochloric acid (HCl, 0.1 M), and sodium hydroxide (NaOH, 0.1 M) were purchased from Merck, India. All reagents and chemicals were used as received without further purification.

### 2.2 Synthesis of the nano La_2_O_3_ adsorbent

Nano lanthanum oxide was synthesized by taking a 1 : 1 molar ratio of lanthanum nitrate, as the precursor, and ammonium solution, as the reducing agent.^39^ Firstly, 0.2 M lanthanum nitrate was prepared in 100 mL of distilled water and stirred for 2 h. At the same time, 0.4 M ammonium solution was prepared in 50 mL of distilled water and then added dropwise to the lanthanum nitrate solution. Slowly, the solution formed a white precipitate and was sonicated for 1 h with a high-density ultrasonic probe immersed directly in the solution. The resulting slurry was collected by centrifugation at 6000 rpm, washed five times with distilled water, and dried overnight at 50 °C in an oven. Then, the dried product was ground and kept in a furnace for 2 h at 300 °C. The stepwise synthesis route of nano La_2_O_3_ is shown in the schematic in Fig. 1.

**Fig. 1:**
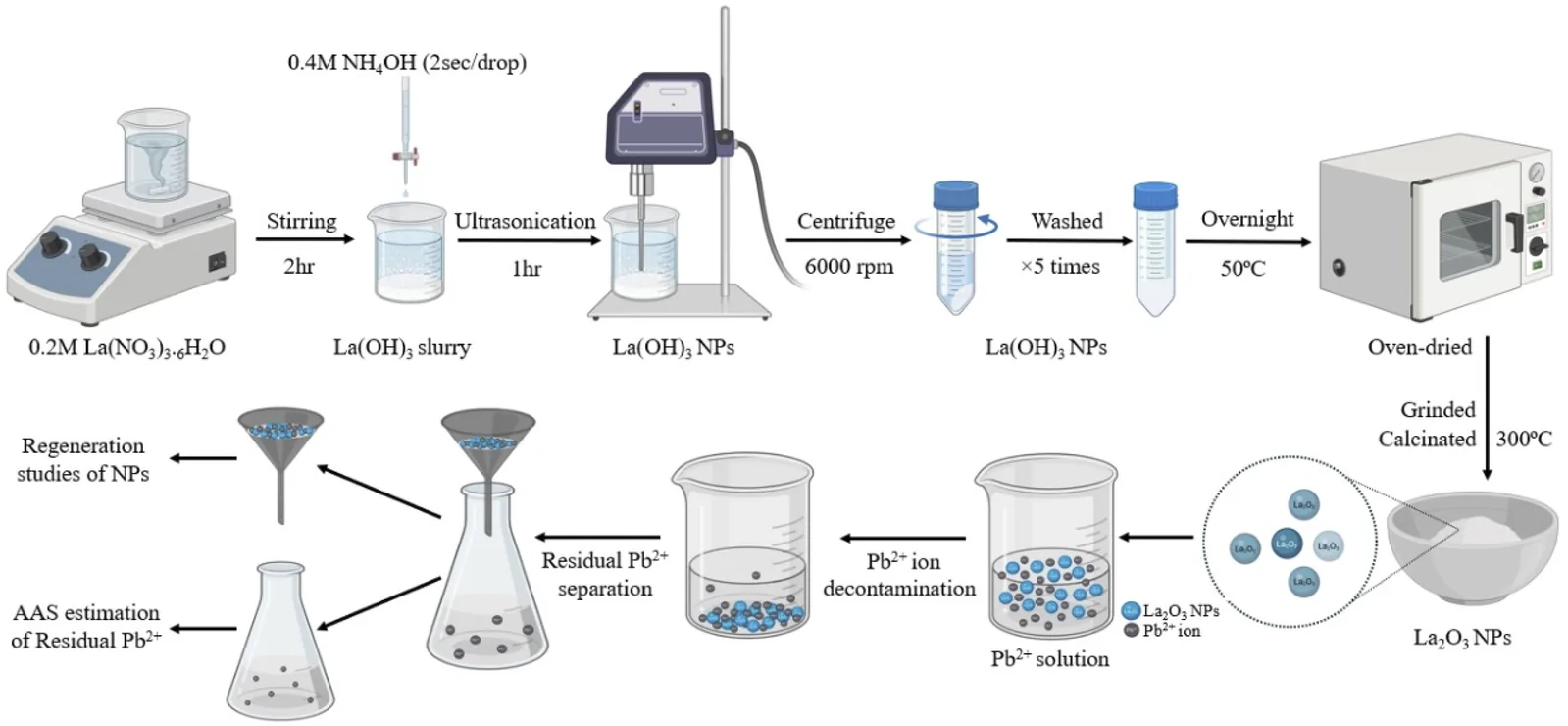
Schematic of the synthesis of nano La_2_O_3_ and its application in the adsorption of aqueous Pb^2+^ ions.

### 2.3 Lead adsorption experiments

The adsorption of lead ions by nano La_2_O_3_ was investigated in a batch experiment. A stock solution of lead was prepared by adding 1.59 g of Pb(NO_3_)_2_ to 1000 mL of distilled water. 1 drop of HNO_3_ was added to keep the stock solution fresh. Lead adsorption experiments were performed by varying the parameters such as the solution pH (2–7), agitation time (2–30 min), temperature (15–45 °C), and adsorbent dosage (0.1–0.5 g L^−1^). All the experiments were conducted at room temperature (25 °C) and a solution pH of 6.0. Generally, 0.2 g L^−1^ nano La_2_O_3_ were added to 100 mL of aqueous lead solutions (50 mg L^−1^) and kept in a rotary shaker (200 rpm) for 60 min to reach the adsorption equilibrium. The solution pH was maintained by adding either 0.1 M NaOH or 0.1 M HCl. After reaching equilibrium, the samples were collected and filtered using Whatman filter paper No. 42. The AAS technique was used to determine the remaining lead concentration. The adsorption capacity for lead uptake “*q*_e_ (mg g^−1^)” was defined as follows:1
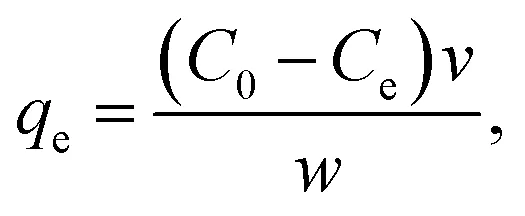
where *C*_0_ and *C*_e_ are the initial and equilibrium concentrations (mg L^−1^) of Pb^2+^ in solution, *V* is the volume (L), and *W* is the weight (g) of the adsorbent. All the experiments were repeated three times under the same experimental conditions. Lead ion adsorption using nano La_2_O_3_ is shown in the schematic in Fig. 1.

### 2.4 Characterization techniques

To determine the atomic and molecular structure of the https://en.wikipedia.org/wiki/Crystal synthesized nano La_2_O_3_, X-ray diffraction (XRD) studies (using a Rigaku D/Max 2200 diffractometer with Cu Kα radiation at *λ* = 1.5406 Å) were carried out. The crystallographic analysis of the samples was performed using diffraction patterns, recorded from 10° to 90° at a counting rate of 1° min^−1^ with an analytical system diffractometer using Cu Kα (*λ* = 1.542 Å) at an accelerating voltage of 40 kV.

To obtain the morphology of nano La_2_O_3_, high-resolution transmission electron microscopy (HRTEM) was carried out using a JEOL JEM-2200FS (Japan) instrument operating at 200 kV. Samples for TEM analysis were prepared by spreading a drop of the prepared dilute dispersion of nano La_2_O_3_ on amorphous carbon-coated copper grids and drying them in air at room temperature.

Scanning electron microscopy (SEM) and energy dispersive X-ray spectroscopy (EDX) were performed to obtain information on the surface topography and composition of nano La_2_O_3_, respectively, using a Joel JSM-6480 LV instrument at an accelerating voltage of 20 kV.

The information on the vibrational states of the adsorbed molecules and the nature of the surface complexes of the synthesized nano La_2_O_3_ was obtained *via* Fourier transform infrared (FTIR) spectroscopy conducted using the Varian FT-Raman and Varian 600 UMA instruments. The samples for determination were prepared by mixing 1% (w/w) specimens with 100 mg of KBr powder and then pressing the mixture into pellets.

A zeta potential analyzer (model ZEECOM-2000, Microtec Co., Ltd, Japan) was used to determine the surface charge of the synthesized nanoparticles. The solutions were diluted to know the surface charge of the synthesized nanoparticles. In the case of interacting solutions, if one uses the zeta potential (*Z*) as an approximation of the surface potential *φ* of a uniformly charged sphere, the theory gives the following relation:2
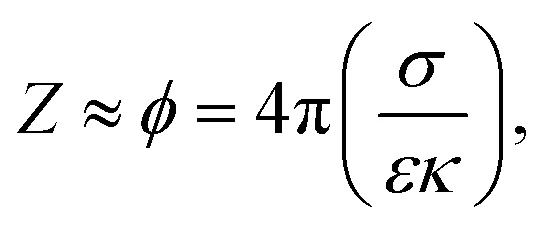
where *σ* is the surface charge density of the particle, and *ε* and *κ* are the dielectric constant and Debye–Huckel parameter of the solution, respectively. The relationship between the mobility (*µ*) and the zeta potential is *Z* = 4π (*µη*/*ε*). Then, *µ* can be written as *µ* = *σ*/*ηκ*, where *η* is the viscosity of the solution.

Atomic absorption spectrometry (AAS) (AAnalyst 400, PerkinElmer) was used to determine lead ions in the water samples. This technique determines the concentration of a particular element (the analyte) in a sample.

## 3 Results and discussion

Fig. 2 shows the XRD pattern of nano La_2_O_3_, which exhibits sharp and intense diffraction peaks corresponding to the (100), (002), (011), (012), (003), (110), (111), (103), (112), (004), (202), (014), (023), (121), and (114) planes at 2*θ* angles of 26.1°, 29.0°, 29.9°, 39.4°, 44.2°, 46.0°, 48.5°, 52.0°, 55.4°, 60.2°, 62.2°, 66.8°, 72.0°, 75.2°, and 79.0°, respectively, confirming the crystalline nature of nano La_2_O_3_. It was confirmed that the synthesized nano La_2_O_3_ was in the hexagonal phase of lanthanum oxide, with the high purity space group *P*3̄*m*1 (164) and lattice parameters of ‘*a*’ = 3.93 Å and ‘*c*’ = 6.136 Å (JCPDS file no. 83-1344).

**Fig. 2:**
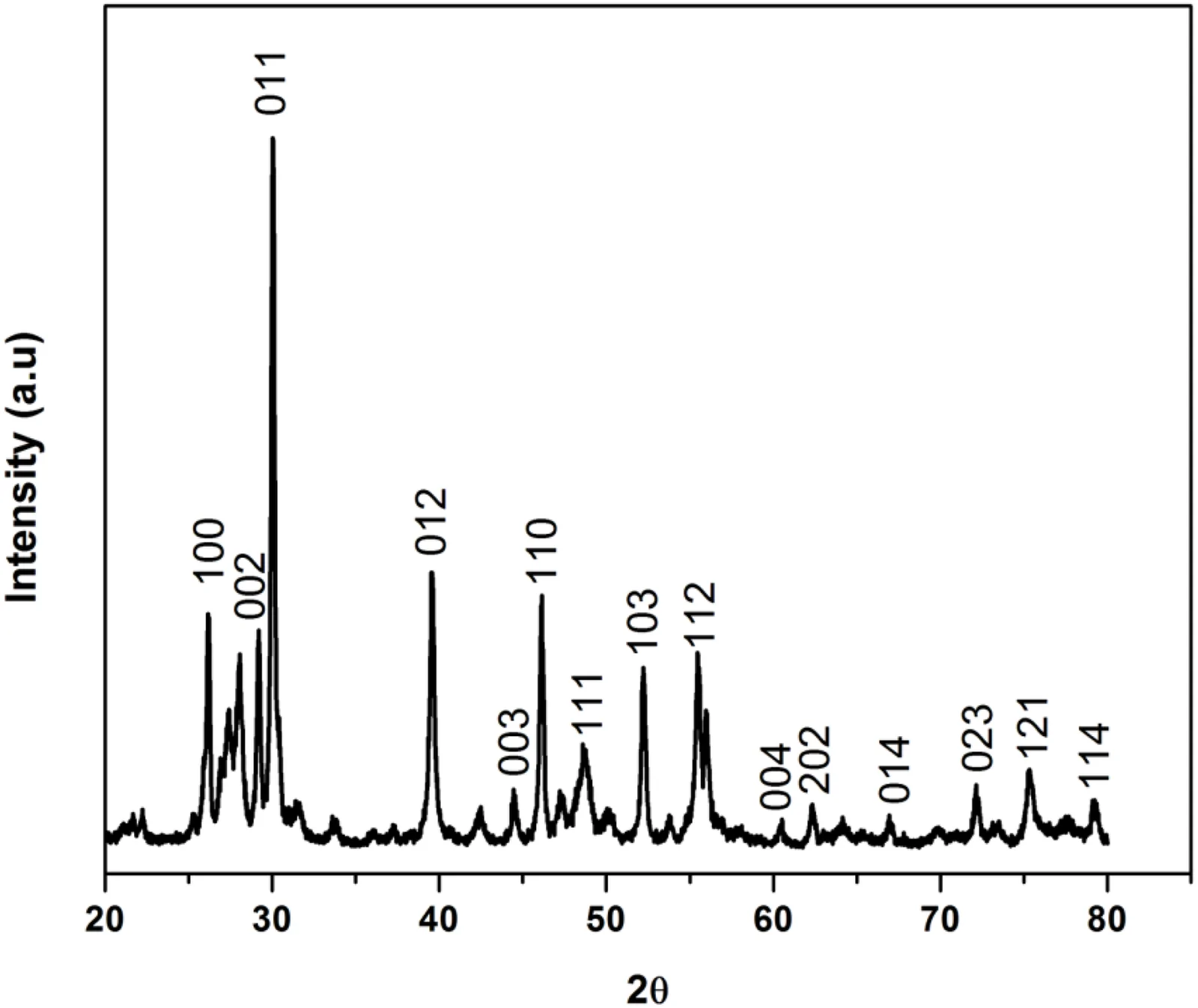
XRD pattern of nano La_2_O_3_.

The particle size of the synthesized NPs was obtained using Scherrer's equation.3
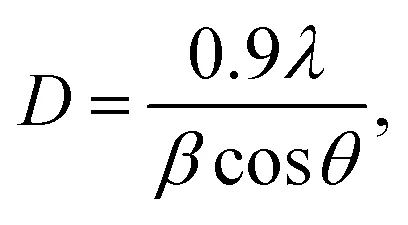
where *D* is the particle size in Å, *λ* is the wavelength of Cu Kα radiation, *i.e.*, 1.54 Å, *β* is the full width at half maximum (FWHM), and *θ* is the angle obtained from 2*θ* corresponding to the maximum peak intensity. The mean crystalline dimension of nano La_2_O_3_ was found to be 14.5 nm.

The TEM and HRTEM images of nano La_2_O_3_ are displayed in Fig. 3(a and b), respectively. It can be clearly seen in Fig. 3(a) that nano La_2_O_3_ exists as monodispersed and irregular spheres with uniform average sizes of 15–25 nm. The XRD diffraction patterns are in close agreement with the TEM results. The HR-TEM images of nano La_2_O_3_ exhibit well-organized lattice planes and interplanar spacing of 0.331 nm [Fig. 3(b)], and the inset shows the SAED diffraction pattern, which reveals the crystalline nature of nano La_2_O_3_.

**Fig. 3:**
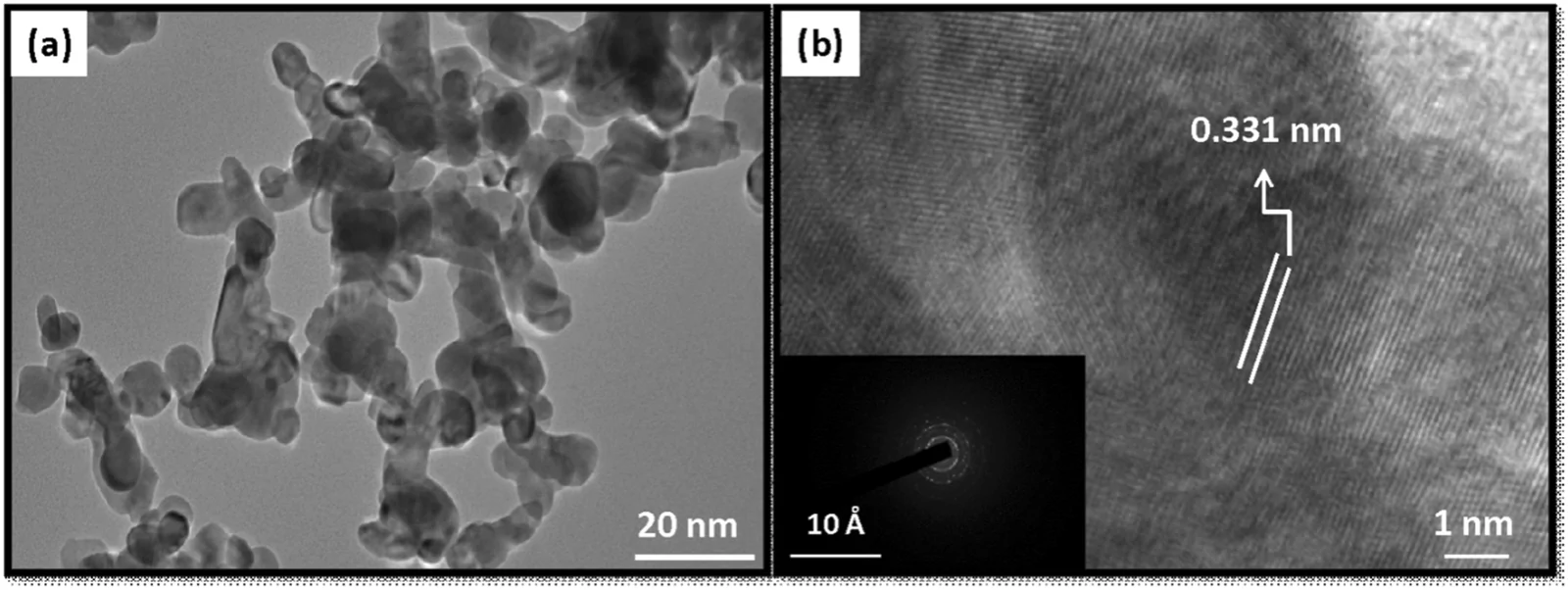
(a) TEM and (b) HRTEM images of nano La_2_O_3_ (inset: SAED diffraction pattern of nano La_2_O_3_, showing the crystalline nature).

Fig. 4(a) shows the SEM image of nano La_2_O_3_. It is clear from the image that the synthesized NPs have globular-shaped structures. The SEM images also show a distinguished spherical morphology with self-aligned prismatic nanoparticles. Fig. 4(b) shows the EDX spectrum of nano La_2_O_3_. EDX analysis reveals the presence of lanthanum (80.02%) and oxygen (19.98%), confirming the purity of lanthanum oxide nanoparticles.

**Fig. 4:**
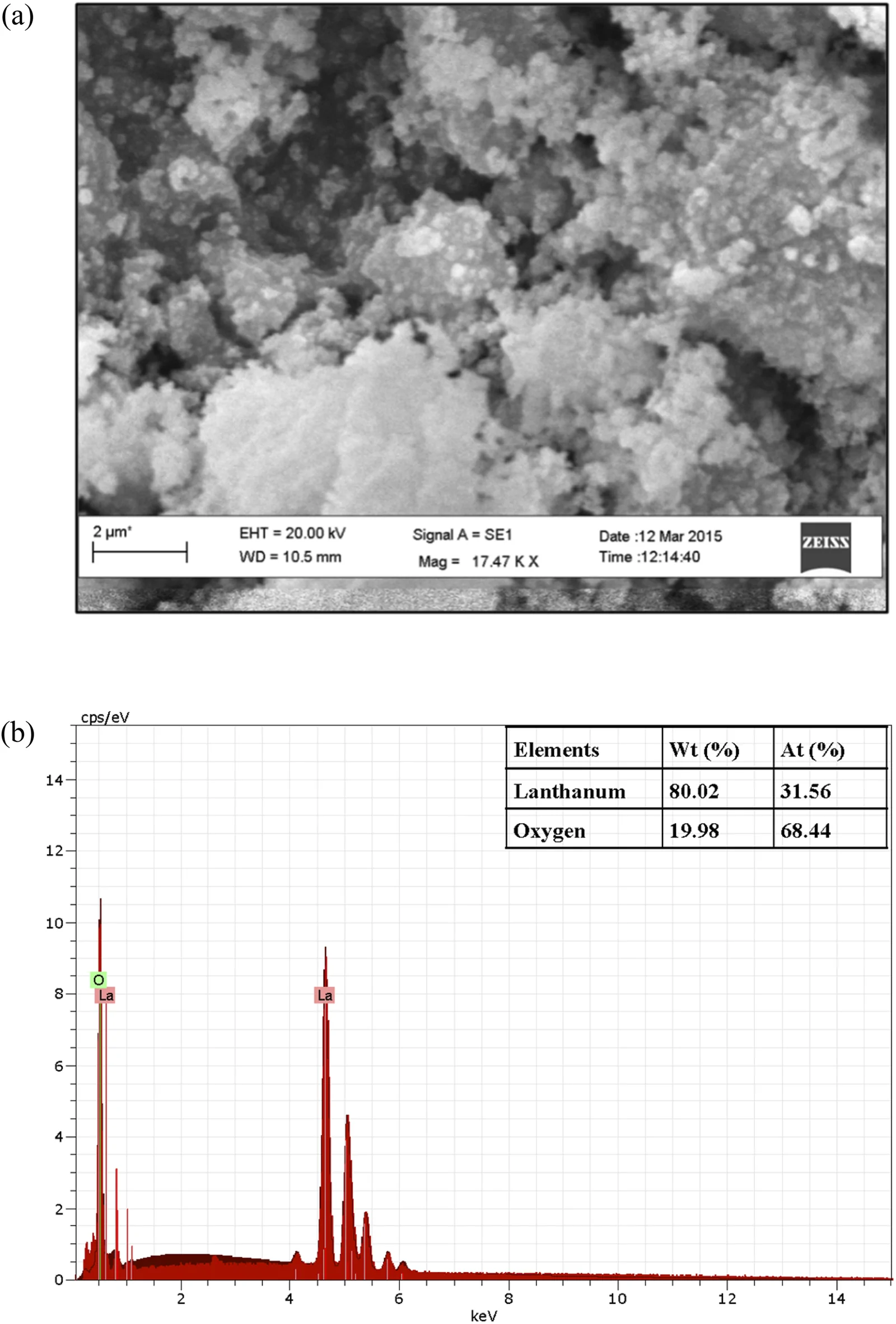
(a)SEM image of nano La_2_O_3_. (b) EDX spectrum of nano La_2_O_3_.

The FTIR spectra of nano La_2_O_3_ were recorded in the 4000–400 cm^−1^ range, as shown in Fig. 5. The absorption band at 3748–3564 cm^−1^ is attributed to O–H bond stretching vibrations and H–O–H bending vibrations from the water molecules on the external surface of the samples during handling.^39–42^ The sharp peak at 3564 cm^−1^ is assigned to the stretching vibrations of O–H in La (OH)_3_.^39,40^. The bands at 3421.4 cm^−1^ and 1642 cm^−1^ can be attributed to the O–H vibrations of the absorbed water on the sample surface.^41,42^ These absorption bands were related to the absorbed water and CO_2_ on the surface of lanthanum hydroxide. A sharp peak was observed at about 462–488 cm^−1^, which can be assigned to the stretching vibration of the La–O bond,^39^ and the band at 650 cm^−1^ is assigned to the bending vibration of La–O–H.^40^ The peak at 2364 cm^−1^ is assigned to the C–O stretching vibration.^40–42^

**Fig. 5:**
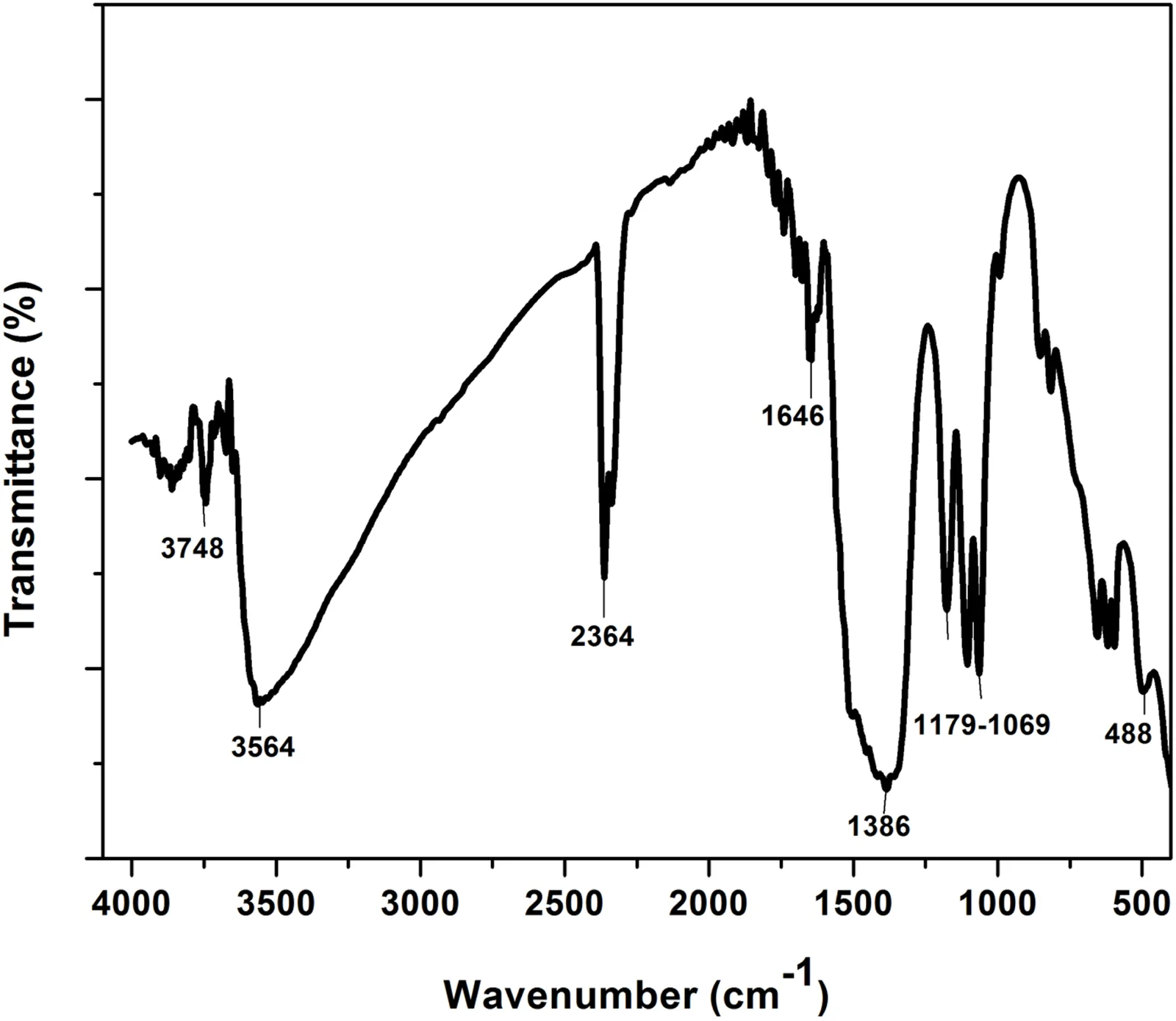
FTIR spectrum of nano La_2_O_3_.

The zeta potential plot of nano La_2_O_3_ across various solution pH levels, from 1 to 7, is illustrated in Fig. 6. The measured zeta potential at pH values ranging from 1 to 7 varied from +10.4 mV to −2.9 mV. The point of zero charge pH (pH_PZC_) for nano La_2_O_3_ was found at a solution pH of 5. At pH values higher than pH_PZC_, nano La_2_O_3_ exhibited a negative zeta potential. Conversely, at pH levels below the pH_PZC_, nano La_2_O_3_ displayed a positive zeta potential, which can be attributed to an increase in the concentration of hydrogen ions (H^+^) due to dissociation. In contrast, at pH levels above pH_PZC_, nano La_2_O_3_ acquired a negative zeta potential as a result of the increased concentration of hydroxide ions (OH^−^).

**Fig. 6:**
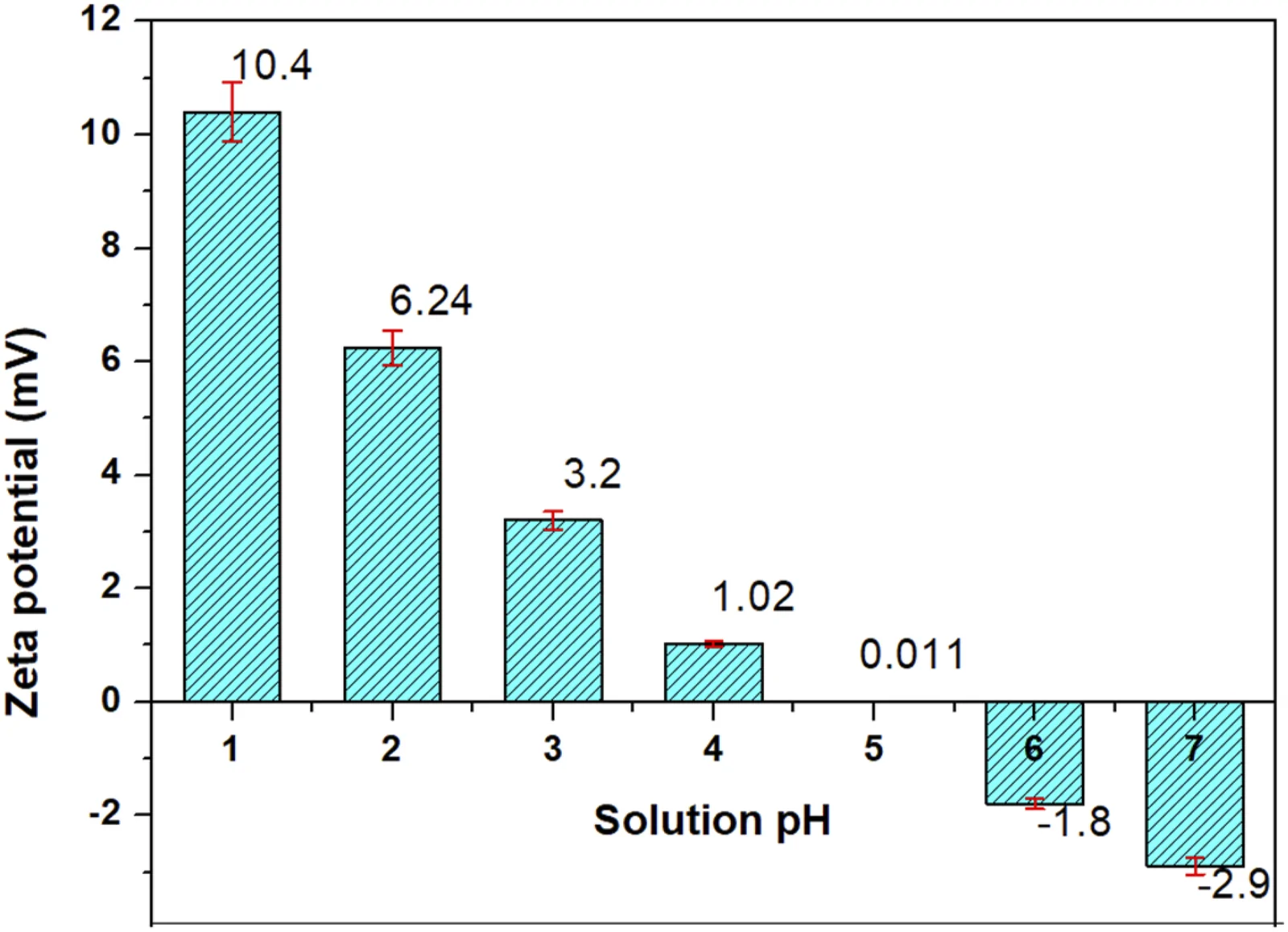
Zeta potential plot of nano La_2_O_3_ at solution pH 1–7.

## 4 Lead (Pb^2+^) adsorption studies

### 4.1 Effect of pH on the adsorption of lead ions

Heavy metal ion adsorption depends on the solution pH due to the surface chemistry in aqueous solutions. The hydroxyl groups of water are bound to the surfaces of metal oxides, and this binding varies with the solution pH.^1,2^ It also creates the adsorbent surface charge and the degree of ionisation and speciation of the adsorbed species.^27,31–35^ The effect of solution pH on the adsorption of Pb^2+^ using nano La_2_O_3_ was conducted at different solution pH of 2 to 7 under optimal experimental conditions (temperature: 25 °C; equilibrium time: 60 min; and agitation speed: 200 rpm) by adding 0.4 g L^−1^ nano La_2_O_3_ in 100 mL of solutions containing an initial Pb^2+^ concentration of 50 mg L^−1^.

Fig. 7 illustrates the impact of the solution pH on the adsorption of Pb^2+^ by nano La_2_O_3_. At a pH of 2, the lead removal efficiency is approximately 36.4%. This efficiency increases as the solution pH rises from 2 to 7, reaching a maximum of 96% at pH 6.0 (pH is greater than the point of zero charge, pH_PZC_ = 5). These results indicate that at higher pH levels (pH > pH_PZC_), the surface of nano La_2_O_3_ carries more negative charge (−1.8 mV at pH 6 and −2.9 mV at pH 7) due to the deprotonation of the surface hydroxyl groups (La–OH + OH^−^ = La–O^−^ + H_2_O), as shown in the zeta potential plot in Fig. 6. This increased negative charge makes the surface more attractive to positively charged Pb^2+^ ions, thereby enhancing lead adsorption (refer to Fig. 6). Conversely, at lower pH levels (pH < pH_PZC_), the nano La_2_O_3_ surface exhibits a net positive charge due to the presence of La_2_–OH_2_^+^ surface groups (+10.4 mV to +1.02 mV), creating a repulsion between the positively charged Pb^2+^ ions and nano La_2_O_3_ (refer to Fig. 6). Similar findings have been reported in earlier studies.^27,31^ The binding mechanism of nano La_2_O_3_ with Pb^2+^ ions is illustrated below. In water, nano La_2_O_3_ forms surface hydroxyl groups, which bind with lead ions, often releasing protons in the process.≡LaOH + Pb^2+^ ↔ ≡LaO − Pb^+^ + H^+^

**Fig. 7:**
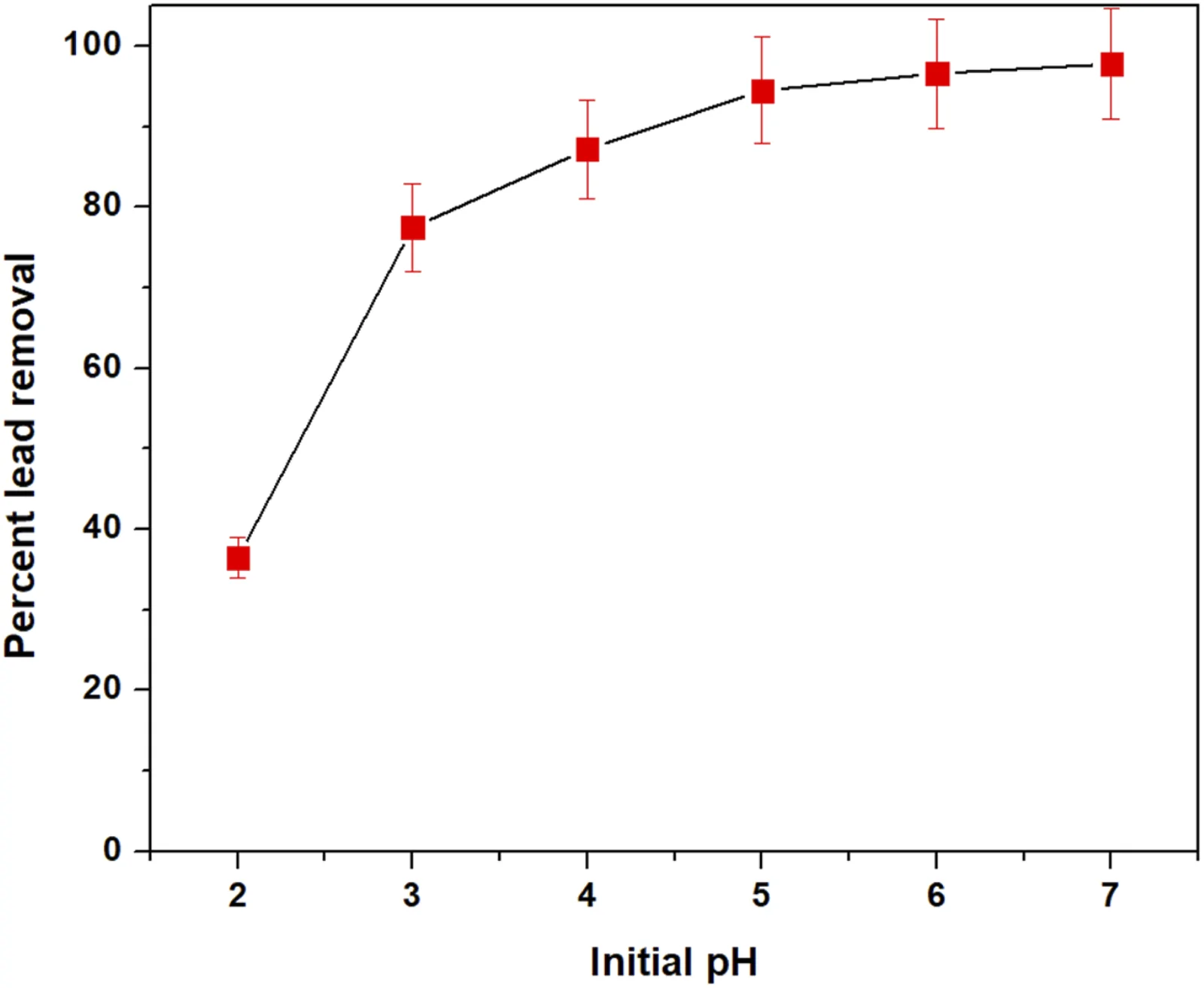
Pb^2+^ sorption curve obtained at different pH values (2, 3, 4, 5, 6 and 7).

### 4.2 Effect of reaction time and kinetic study

To obtain the optimum contact time, the rate of adsorption of Pb^2+^ on nano La_2_O_3_ was studied at different time intervals (2, 4, 6, 10, 15, 20, 25, and 30 min) under optimal conditions (pH: 6; adsorbent dose: 0.2 g L; Pb^2+^ concentration: 50 mg L; temperature: 25 °C; and agitation speed: 200 rpm) [Fig. 8]. The lead removal efficiency increased from 35% to 98% with an increase in time from 2 to 30 min. Almost 64% of Pb^2+^ uptake occurred within 10 min, and 97% of Pb^2+^ uptake was completed within 25 min. The rate was initially rapid and then slowed down as equilibrium approached. The adsorption equilibrium was achieved within 25 min. This observation shows the rapid adsorption of Pb^2+^ onto nano La_2_O_3_; this may be attributed to the external surface adsorption and size of nano La_2_O_3_.

**Fig. 8:**
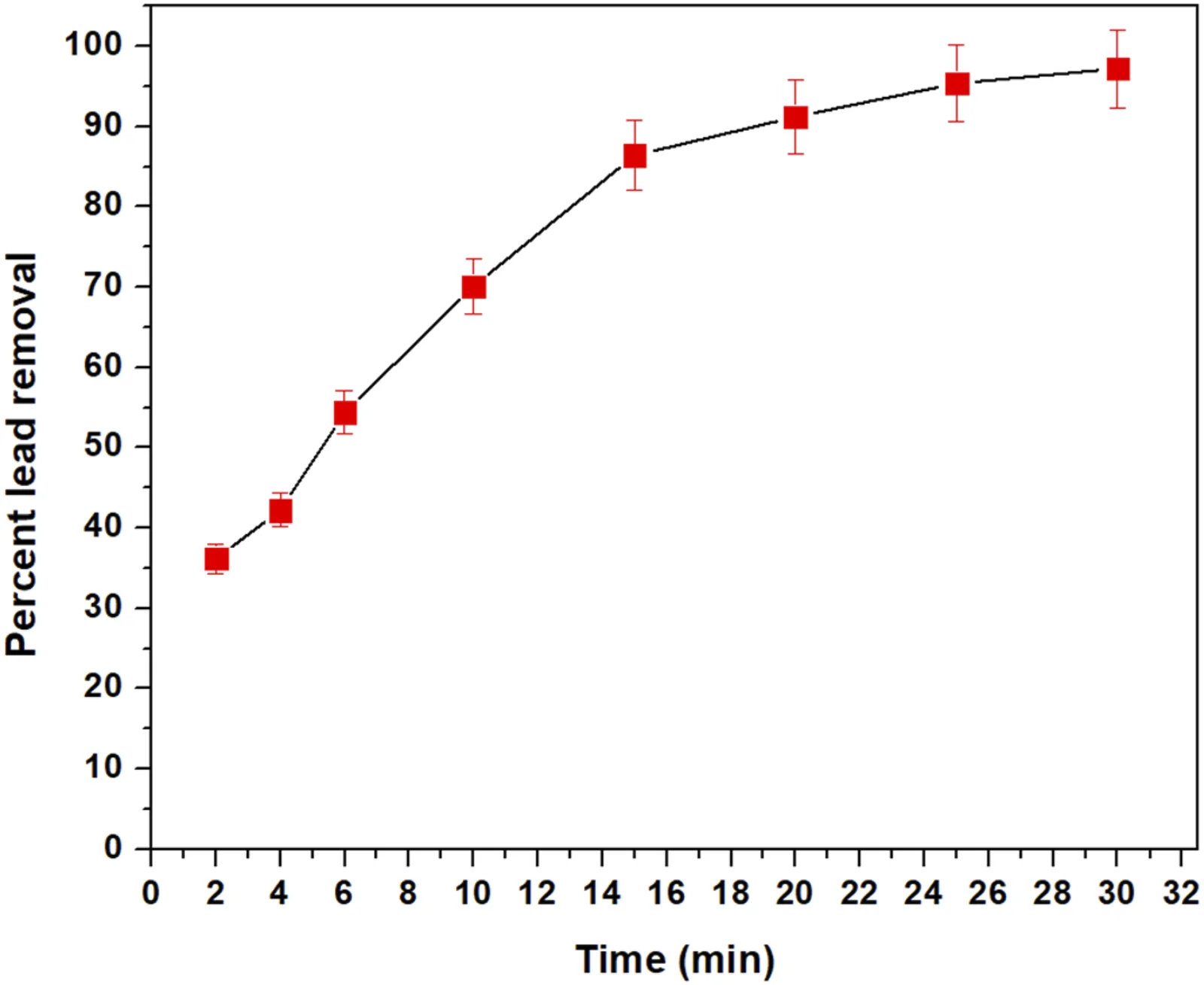
Pb^2+^ sorption curve obtained at different time intervals (2, 4, 6, 10, 15, 20, 25, and 30 min).

**Fig. 9:**
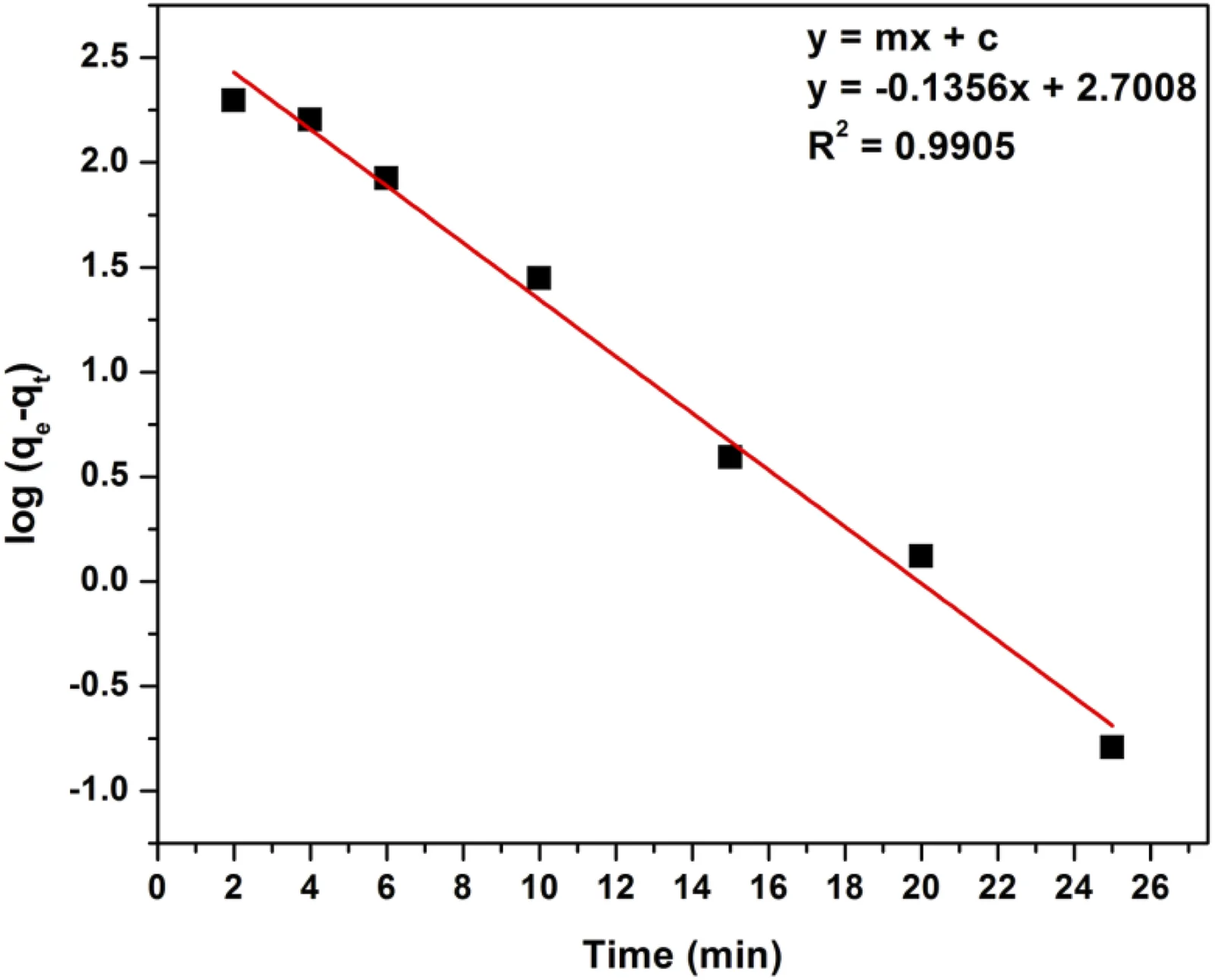
Pseudo-first-order kinetic plot for Pb^2+^ adsorption onto nano La_2_O_3_.

The pseudo-first-order and pseudo-second-order kinetic models were applied to determine the mechanisms of Pb^2+^ adsorption onto nano La_2_O_3_.^43,44^Fig. 9 and 10 show the pseudo-first-order and second-order kinetic model plots, showing the variation in the amount of adsorbed species (*q*_*t*_) as a function of time. The slope and intercept were obtained from the plots, and the coefficient of determination (*R*^2^) and kinetic rate constant (*k*) were calculated using eqn (4) and (5). The obtained kinetic parameters, *i.e.*, the coefficient of determination (*R*^2^) and kinetic rate constant (*k*), are illustrated in Table 1. The highest coefficient of determination (*R*^2^ = 0.9921) was obtained for the pseudo-second-order model, suggesting it to be the best fit model. This indicates that the adsorption of Pb^2+^ ions onto nano La_2_O_3_ is controlled by chemisorption.^1,2^ Thus, the Pb^2+^ adsorption rate depends on the number of La_2_O_3_ surface sites. In the linear form of these two models, the equations can be written as follows:4
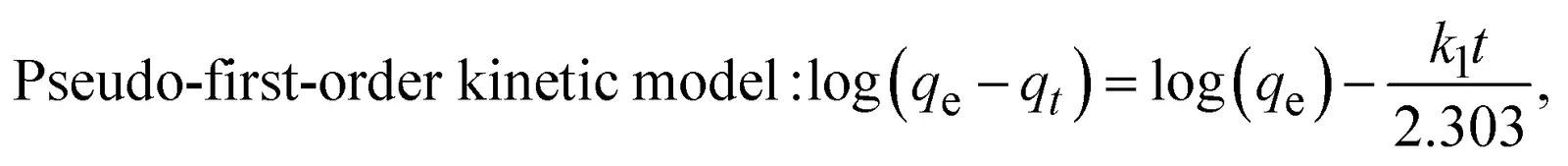
5
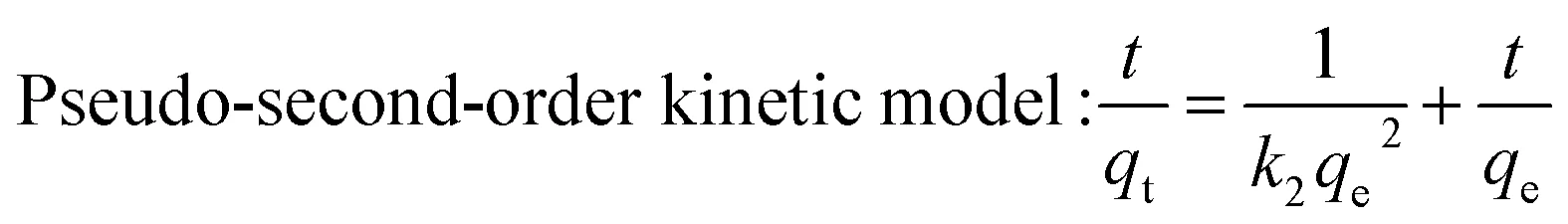


**Table 1:** Pseudo-first-order and pseudo-second-order rate constants and correlation coefficients obtained for Pb^2+^ adsorption onto nano La_2_O_3_

Kinetic model parameter	Pseudo-first-order	Pseudo-second-order
Slope (*m*)	−0.1356	0.0523
Rate constant (*k*) unit	0.1356	0.0086
Intercept	2.7008	0.3153
*q*_e_ (mg g^−1^) (equilibrium adsorption capacity)	14.89	19.12
*R* ^2^	0.9905	0.9921

The product *k*_2_*q*_e_^2^ is the initial sorption rate. *k*_1_ and *k*_2_ (min^−1^) are the first-order and second-order adsorption rate constants, respectively, *q*_e_ is the amount of lead adsorbed at equilibrium, and *q*_*t*_ is the amount of lead absorbed at time ‘*t*’ (Fig. 10).

**Fig. 10:**
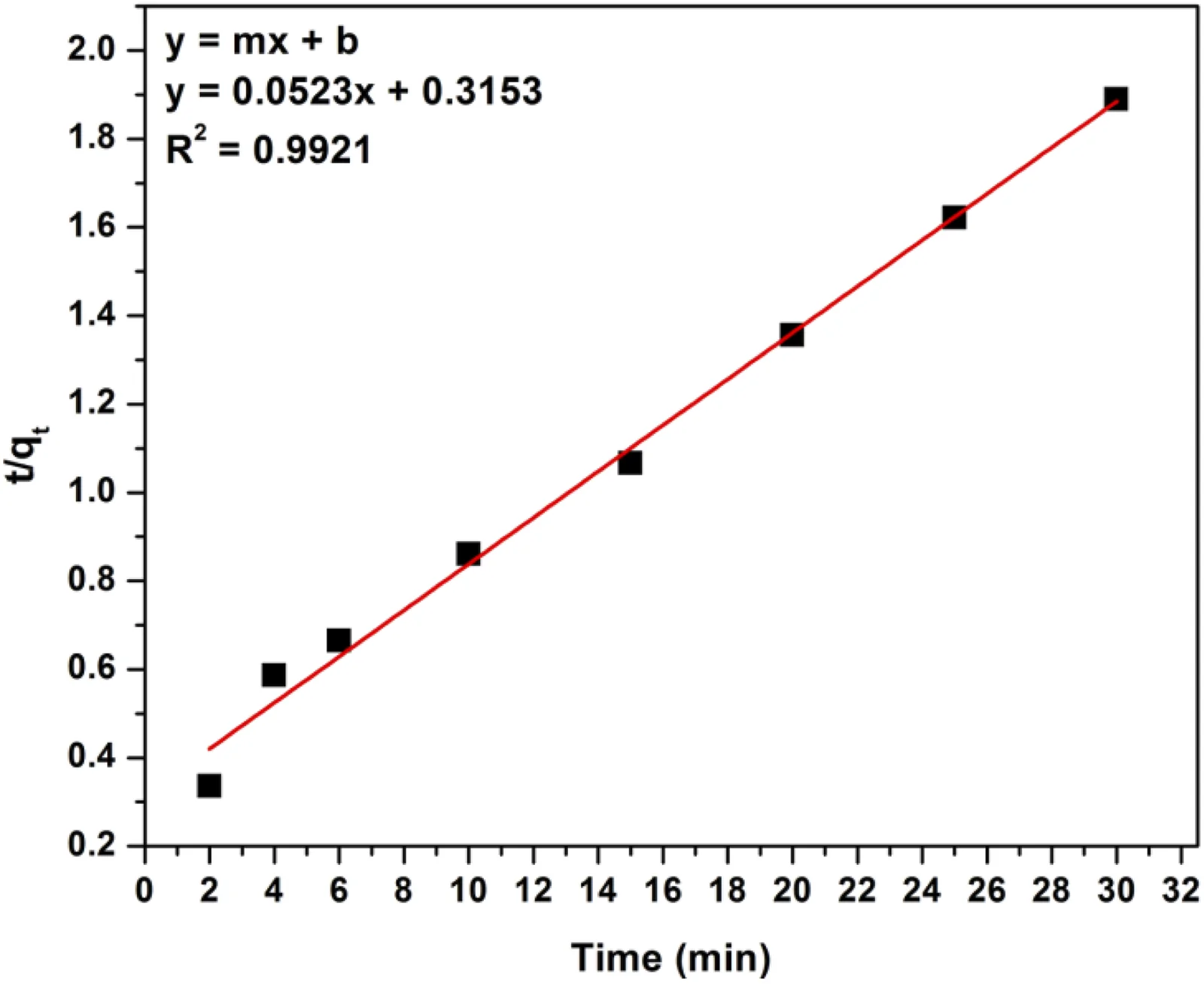
Pseudo-second-order kinetic plot for Pb^2+^ adsorption onto nano La_2_O_3_.

### 4.3 Effect of nano La_2_O_3_ dosage

The influence of the adsorbent (nano La_2_O_3_) dosage on Pb^2+^ adsorption was studied by varying the NP dose as 0.1, 0.2, 0.3, 0.4 and 0.5 g L^−1^ (Fig. 11). The experiment was conducted at an initial Pb^2+^ concentration of 50 mg L^−1^ in 100 mL of a solution under optimum conditions (pH: 5, temperature: 25 °C, agitation speed: 200 rpm, and equilibrium time: 60 min). The Pb^2+^ adsorption increased substantially from 38% to 99.9% with an increase in the nano La_2_O_3_ dosage (0.1–0.5 g L^−1^). An increased adsorbent dosage implied a greater surface area and a more significant number of available binding sites for the adsorption of lead ions.^46^ Therefore, as shown in Fig. 11, the residual lead concentration gradually decreased with an increase in the adsorbent dose. The equilibrium region of Pb^2+^ adsorption was reached at 0.4 g L; there was no further binding after that.

**Fig. 11:**
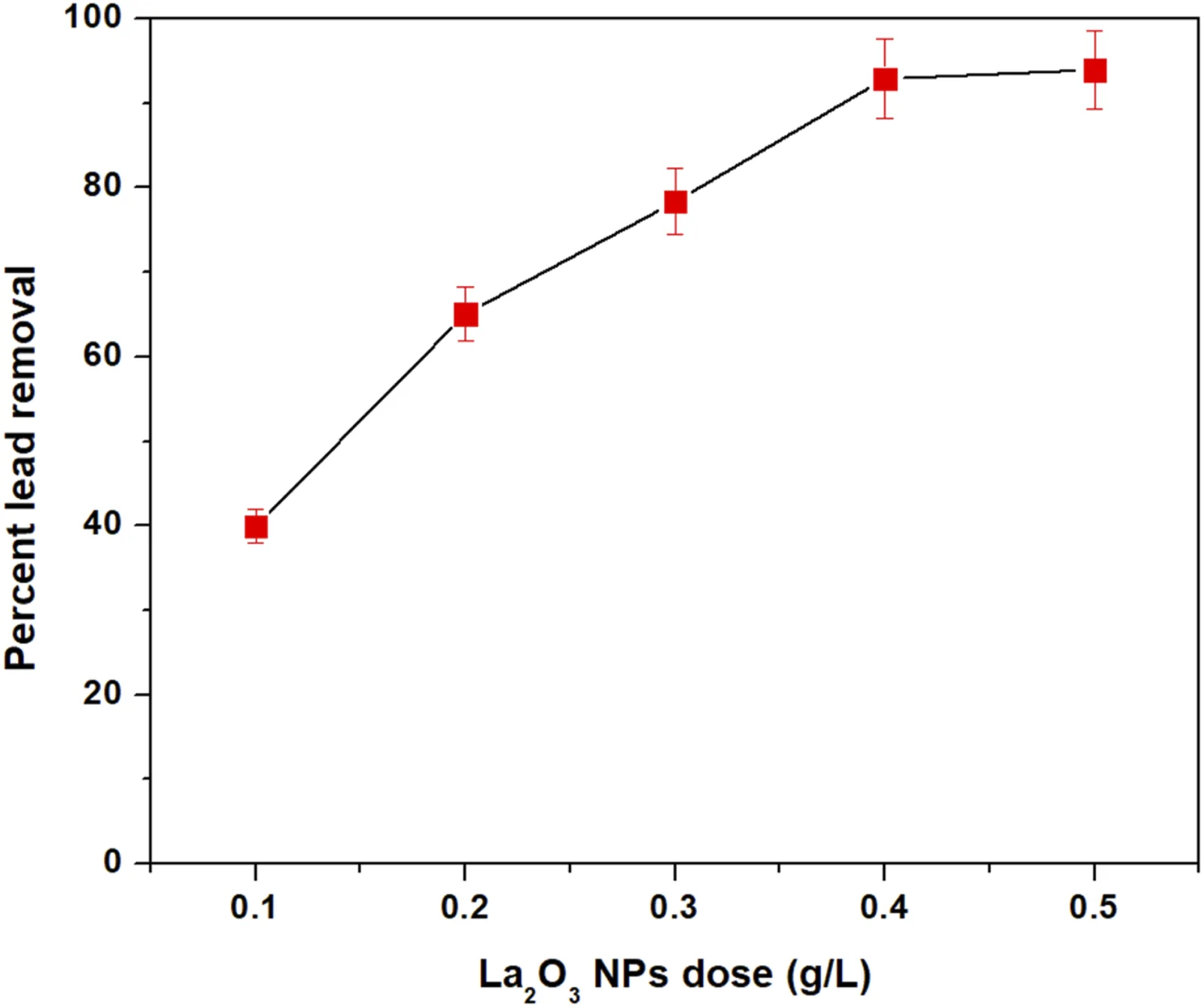
Pb^2+^ sorption curve obtained at different doses of nano La_2_O_3_ (0.1, 0.2, 0.3, 0.4 and 0.5 g L^−1^).

### 4.4 Effect of temperature on lead adsorption

To examine the effect of temperature on the adsorption of Pb^2+^ onto nano La_2_O_3_, experiments were carried out at temperatures of 15 °C, 25 °C, 35 °C, and 45 °C under optimal conditions (pH: 5, nano La_2_O_3_: 0.4 g L^−1^, Pb^2+^ conc.: 50 mg L^−1^, equilibrium time: 60 min, and agitation speed: 200 rpm). It is clear from Fig. 12 that Pb^2+^ adsorption increased with an increase in temperature. This indicates that temperature influences the adsorption capacity, suggesting that the process is endothermic. The increase in Pb^2+^ adsorption with increasing temperature is probably due to a combination of the enhanced diffusion and surface area growth of the adsorbent caused by widening and deepening of the tiny microspores, thereby creating more sites for adsorption.^35,36^ Thus, the increase in the number of adsorption sites due to the breaking of some internal bonds near the edge of the particles leads to higher adsorption of Pb^2+^, thereby increasing the removal efficiency of Pb^2+^ from aqueous solutions.^37,39^

**Fig. 12:**
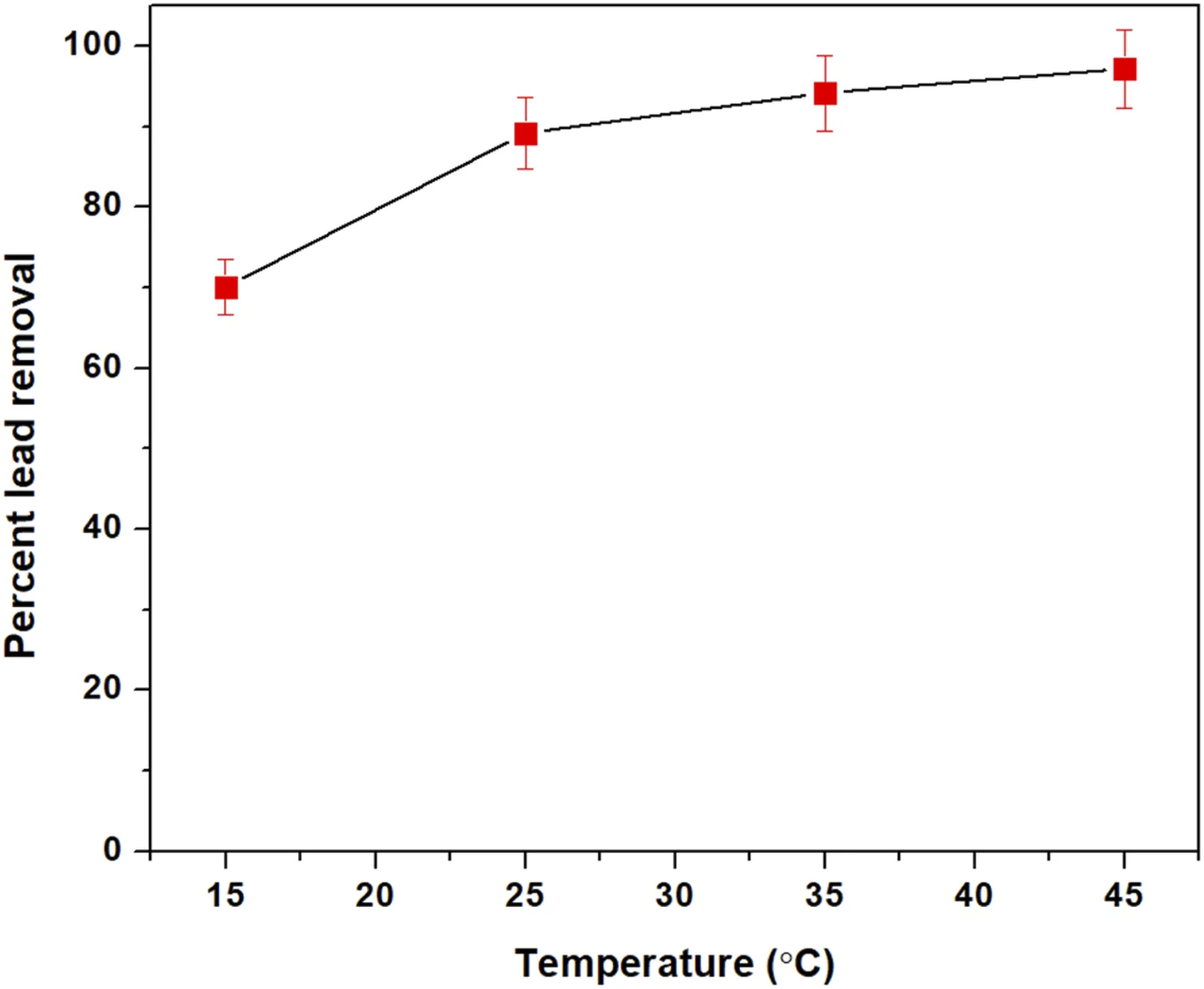
Pb^2+^ sorption curves obtained at different temperatures (15 °C, 25 °C, 35 °C and 45 °C).

## 5 Regeneration studies of spent nano La_2_O_3_

The desorption process of the spent nano La_2_O_3_ was conducted using a 0.05 M HNO_3_ solution as the desorbing agent through a 5-cycle method.^43^ In this experiment, 0.1 g L^−1^ nano La_2_O_3_ was added to 100 mL of a solution containing 50 mg L^−1^ Pb^2+^ ions. The solution was agitated for 60 minutes at a shaking speed of 200 rpm. To regenerate, the spent nano La_2_O_3_ was desorbed using 0.05 M HNO_3_ for five cycles. As illustrated in Fig. 13, approximately 88% of the total lead was recovered in the first cycle (20 mL), while about 58.2% was desorbed in the second cycle. The remaining lead was desorbed in three additional 20 mL increments of 0.05 M HNO_3_. These results indicate the successful desorption of nano La_2_O_3_, enabling its reuse and making it economically viable.

**Fig. 13:**
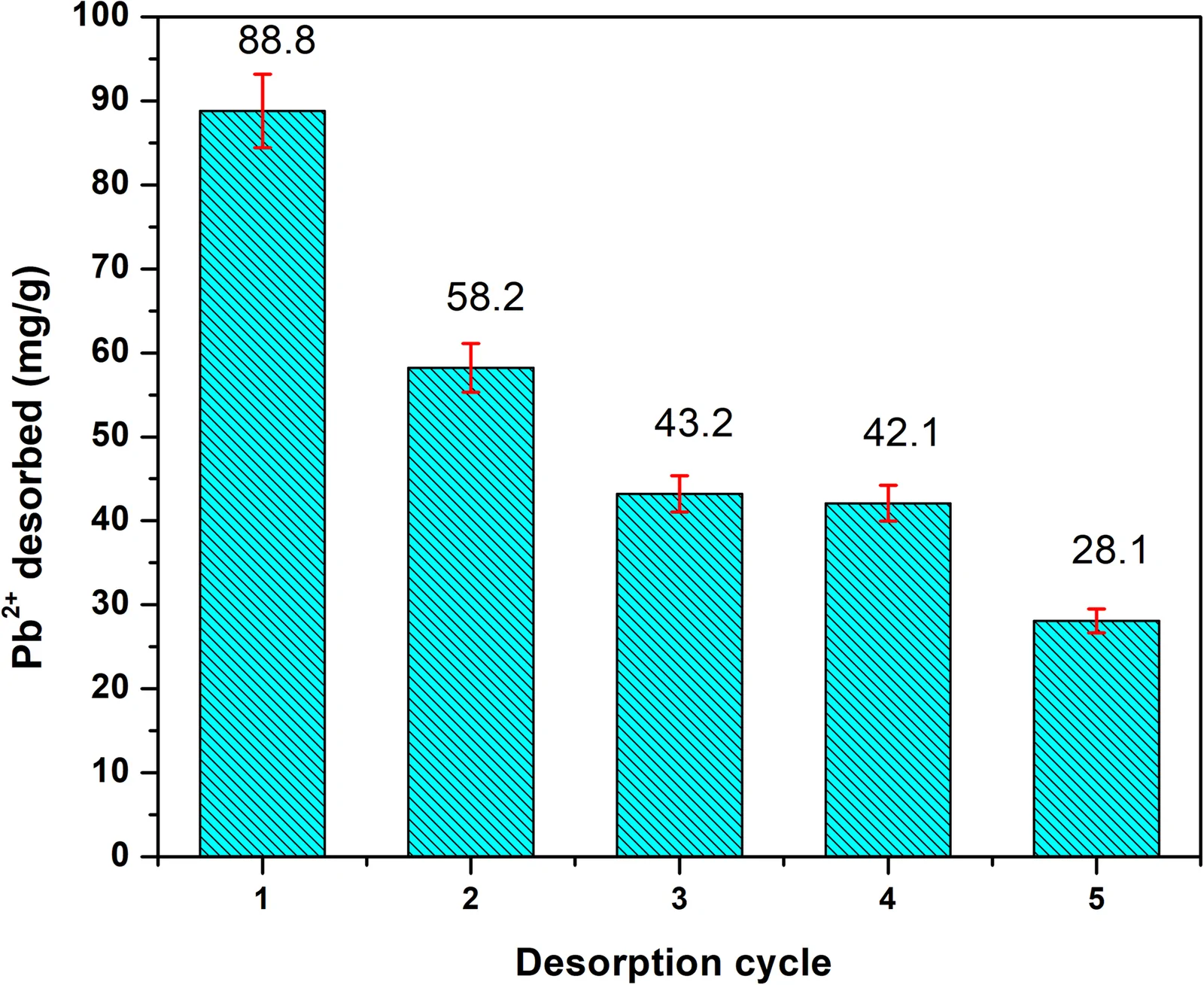
Regeneration of spent nano La_2_O_3_ using 0.05 M HNO_3_ as the desorption agent.

## 6 Conclusions

Nano La_2_O_3_ was successfully synthesized using a simple sonication method and characterized using various techniques. These nanoparticles were effective in removing lead ions from water in a rapid manner. Remarkably, nearly 98% of lead was eliminated from water within 25 minutes at a solution pH of 6. The results aligned well with the pseudo-second-order kinetic model, indicating that the adsorption of Pb^2+^ ions onto nano La_2_O_3_ occurred *via* chemisorption. The rate of equilibrium adsorption capacity (*q*_e_, mg g^−1^) of lead uptake was obtained to be 19.12 mg g^−1^. Thermodynamic studies revealed that lead adsorption onto nano La_2_O_3_ is an endothermic process. The 5-cycle desorption studies were successfully performed to regenerate spent nano La_2_O_3_ using HNO_3_ (0.05 M) as the desorbing agent, thereby enabling stability and reuse. Therefore, nano La_2_O_3_ is recommended as a fast, efficient, reusable, and biocompatible nano-adsorbent for the rapid removal of lead ions from contaminated water.

## Conflicts of interest

There are no conflicts to declare.

## Data Availability

The authors confirm that the data supporting the findings of this study are available within the article. Raw data that support the findings of this research article are available from the corresponding authors upon reasonable request.
